# Supplementary material for: Commitment to protective measures during the COVID-19 pandemic in Syria: A nationwide cross-sectional study
Source: PLoS One. 2022 Oct 14;17(10):e0275669. doi: 10.1371/journal.pone.0275669 (PMC9565665; doi:10.1371/journal.pone.0275669)
Supplement: Attachment — Submitted filename: Response to Reviewers.docx [file pone.0275669.s002.docx]

Response letter

**We would like to thank the editor and reviewers for taking their valuable time to review this manuscript. We have thoroughly assessed the comments and implemented them into our revised manuscript, now that this article has been made suitable we aspire to have this article published in your journal.**

**The following letter will report the responses to the reviewers’ comments.**

**Journal Requirements:**

1. Please ensure that your manuscript meets PLOS ONE's style requirements, including those for file naming. The PLOS ONE style templates can be found at

**Author response:** All style requirements have been addressed, including those for file naming.

2. Your ethics statement should only appear in the Methods section of your manuscript. If your ethics statement is written in any section besides the Methods, please delete it from any other section.

**Author response:** We apologize for this mistake and have deleted The ethical statement from the other sections.

3. Please review your reference list to ensure that it is complete and correct. If you have cited papers that have been retracted, please include the rationale for doing so in the manuscript text, or remove these references and replace them with relevant current references. Any changes to the reference list should be mentioned in the rebuttal letter that accompanies your revised manuscript. If you need to cite a retracted article, indicate the article’s retracted status in the References list and also include a citation and full reference for the retraction notice.

**Author response:** The reference list have been revised thoroughly.

**Review Comments to the Author**

**Reviewer #1:** In this paper entitled "Commitment to protective measures during COVID-19 pandemic in Syria: a nationwide cross-sectional study", the authors evaluate the extent to which the Syrian population adheres to these measures and analyze the relationship between demographic variables and adherence. The manuscript is well written and intelligently presented. The study is nicely designed, and the sample size is calculated using OpenEpi online software. All the data is analyzed using Statistical Package for Social Sciences version 25.0. The results are presented in a tabular form that gives a complete overview of the data collected. In addition, percentage, correlation and p-value are calculated to provide statistical stringency to the manuscript. I congratulate the authors for this work and recommend this work for publication. The manuscript is suitable for publications and should be formally accepted once it meets all technical requirements. Although it has one or two typos in the manuscript (lines 229,230),it needs to provide high-resolution fig 1 or remove it from the manuscript.

**Author response:** We apologize for this mistake and have revised this manuscript thoroughly for any other typos. We also provided a high-resolution copy of figure 1.

**Reviewer #2:** The manuscript entitled “Commitment to protective measures during COVID-19 pandemic in Syria: a nationwide cross-sectional study.” written by Shibani et al investigated the extent to which the Syrian population adheres to precautionary infection control measures and analyze the relationship between demographic variables and adherence. Authors did a cross-sectional study in Syria between January 17 and March 17, 2021 using a structured self-administered questionnaire. Authors concluded that this study generally showed a high level of adherence to the preventive measures compared to participants in other studies from around the world, with some concerns regarding risk perception and the sources of information they depend on. This manuscript requires minor revision prior to its publication in PLOS One as follows:
Suggestions:

1. The background section can be updated with minor information such as health, diet and immunity, the natural COVID-19 preventive measures and various variants of COVID-19 i.e. doi: 10.1007/s12088-020-00893-4; doi: 10.1007/s12088-020-00908-0; doi: 10.1007/s15010-021-01734-2.

**Author response:** We thank the reviewer for the guidance, and have used the valuable information provided above to enhance our paper quality. These amendments have been made to the manuscript text and where these can be viewed (Background section, lines 67-68)

2. Figures quality may be improved with high resolution images (minor).

**Author response:** We apologize for the inconvenience and have provided a high-resolution copy of figure 1.

3. It would be required to provide one illustrative Figure as to highlight the summary or future prospect of this study.

**Author response:** We thank the reviewer for this comment, and would like to ask for further instructions about how to address this point.

4. Did authors ask about vaccination status in their survey (vaccinated/not vaccinated/will receive vaccination/reluctant for vaccination?

**Author response:** We thank the reviewer for this comment, and would like to clarify that at the time when this study was conducted, no vaccine were available in Syria, so our main focus was to study the commitment to personal protective measures regardless of the availability of the vaccines. However, there is another study was conducted at the same period regarding this manner i.e.: <https://doi.org/10.1186/s12889-021-12186-6>

5. The English of manuscript can be polished (minor).

**Author response:** We apologize for this mistake and have revised this manuscript thoroughly for language editing.

6. The authors should cross-check all abbreviations in the manuscript. Initially, define in full name followed by abbreviation.

**Author response:** We apologize for this mistake and have revised this manuscript thoroughly for abbreviations.

**Reviewer #3:** This study investigated the commitment status of Syrian population to the protective measures against SARS-CoV-2 viral infection during COVID-19 pandemic. The manuscript is well organized and worth to be published. Yet, given the high contagious nature of the SARS-CoV-2 virus, commitment to the protective measures may only help to delay the infection to some limited extent, and eventually all members of the public will get infected by the virus in the long time. So, the paper will be more attractive if the authors can also provide some insightful understanding about infectious diseases like the SARS-CoV-2 infection, so that the public know what to do to avoid to develop into severe case of COVID-19 if they are infected by SARS-CoV-2 virus.

In talking about infectious diseases, it would be better if we distinguish infections from diseases. Infection is the presence of pathogen, which is not a sufficient condition for disease. [1] Like most of the other viral and bacterial infections, the SARS-COV-2 viral infection is self-limiting [2]. When the human cells are infected by virus or bacteria, the human immunity will actively induce cell self-destruction (programmed cell deaths like necroptosis [3] and pyroptosis [4]) to stop the intracellular infection and reuse the nutrition from the degradation of these destroyed cells and the microorganisms in these cells to rebuild the tissue cells [5]. So by actively destroy damaged somatic cells, the viral and bacterial infections are also removed and become self-limiting [6]. Therefore most of the COVID-19 cases are asymptomatic or very mild. Most of the severe cases of infectious diseases are the result of the overreaction of the strong yet malfunctioning immune system, as Sir William Osler stated more than 100 years ago: "Except on few occasions, the patient appears to die from the body’s response to infection rather than from it. Sir William Osler (1904)" [7]. As the human immune system also plays a virtual role in nutrition acquisition from the degradation of viral damaged epithelial cells, the nutrition surge coupled with the overnutrition state in some patients with obesity or metabolic syndromes may contribute to lipotoxicity and damage in non-adipose tissues, triggering hyperinflammation and contribute to the malfunction of the immune system. So the SARS-CoV-2 viral infection is only the trigger for the COVID-19 disease, and the real cause for the severe cases of COVID-19 disease are autoimmune disorder [8] caused by overnutrition which leads to the hyperinflammation and cytokine storm in these patients.

References:

1. V Humphries DL, Scott ME, Vermund SH (2021) Pathways linking nutritional status and infectious disease: causal and conceptual frameworks. In: Nutrition and infectious diseases, Shifting the Clinical Paradigm (eds DL Humphries, ME Scott, SH Vermund), pp. 3–22. Cham, Switzerland: Humana. DOI: 10.1007/978-3-030-56913-6_1
2. Kumar S, Veldhuis A, Malhotra T (2021) Neuropsychiatric and Cognitive Sequelae of COVID-19. Front Psychol 12:577529. DOI: 10.3389/fpsyg.2021.577529.
3. Nailwal, H., Chan, F.KM. Necroptosis in anti-viral inflammation. Cell Death Differ 26, 4–13 (2019). DOI: 10.1038/s41418-018-0172-x
4. Jorgensen I, Miao EA (2015) Pyroptotic cell death defends against intracellular pathogens. Immunological Reviews. 265 (1): 130-142. DOI:10.1111/imr.12287
5. Maitre Y, Mahalli R, Micheneau P, Delpierre A, Amador G, Denis F (2021) Evidence and Therapeutic Perspectives in the Relationship between the Oral Microbiome and Alzheimer's Disease: A Systematic Review. Int J Environ Res Public Health 18(21):11157. DOI: 10.3390/ijerph182111157.
6. Levin BR, Baquero F, Ankomah P, McCall IC (2017) Phagocytes, Antibiotics, and Self-Limiting Bacterial Infections. Trends in Microbiology, 25(11):878-892. DOI: 10.1016/j.tim.2017.07.005
7. Dobson GP, Biros E, Letson HL and Morris JL (2021) Living in a Hostile World: Inflammation, New Drug Development, and Coronavirus. Front. Immunol. 11:610131. DOI: 10.3389/fimmu.2020.610131
8. Halpert G, Yehuda Shoenfeld Y (2020) SARS-CoV-2, the autoimmune virus. Autoimmunity Reviews 19(12):102695. DOI: 10.1016/j.autrev.2020.102695.

**Author response:** We really appreciate the efforts made by the reviewer and have used his/her guidance to further improve our manuscript. and we hope that the study is now better than before These amendments have been made to the manuscript text and where these can be viewed (Background section, line 69-76, and Discussion section, line 240-249)
