# Supplementary material for: Commitment to protective measures during the COVID-19 pandemic in Syria: A nationwide cross-sectional study
Source: PLoS One. 2022 Oct 14;17(10):e0275669. doi: 10.1371/journal.pone.0275669 (PMC9565665; doi:10.1371/journal.pone.0275669)
Supplement: Attachment — Submitted filename: Response letter.docx [file pone.0275669.s003.docx]

Response letter

**We would like to thank the editor for taking the valuable time to review this manuscript. We have thoroughly assessed the comments and implemented them into our revised manuscript, now that this article has been made suitable, we aspire to have this article published in your journal.**

**The following letter will report the responses to the comments.**

**Editor comments:**

- Title: Please add ‘the’ before COVID-19 pandemic.

**Author response:** We thank the editor for the guidance, and have made the asked amendment

Abstract:

1- Please report the sample size and sample type in the methods section of abstract.

**Author response:** We thank the editor for the guidance, and have made the asked amendment. These amendments have been made to the manuscript text and where these can be viewed (Abstract section, line 34-35)

2- Please report the total number of respondents and the response rate in the results section.

**Author response:** the total number of respondents and the response rate have been added in the results section, which can be viewed in (Abstract section, line 38)

3- Please revise this sentence as it is not clear “Statistically significant differences across age, sex, marital status, financial status, employment, and educational attainment when correlated against commitment to preventive measures.”

**Author response:** We thank the editor for the guidance, and have revised this sentence and made it more clear. This can be viewed (Abstract, line 41-44)

4- Please reconcile your statements about adherence to preventive measures in the abstract because in the results section you report ‘good adherence ‘while in the conclusion section you state ‘high level of adherence’. Good and High level are not same thing. Moreover, you have reported ‘Low’, ‘Moderate’ and ‘High’ categories of commitment in the methods section (lines 155-156). Therefore, please be consistent and report the level of commitment that was found.

**Author response:** We understand the editor’s point, and have revised the manuscript to ensure we have used the same term and classification in all sections, and correction to the mentioned sentence were made. these can be viewed (Abstract, line 40 -41)

Background:

1- Could you please report the date on which there were ‘…over 250 million cases and over 5 million deaths (1)’

**Author response:** We apologize for this mistake, and have reported the date. These amendments have been made to the manuscript text and where these can be viewed (Background, line 57-58)

2- Please refer to your statement about the possible impact of nutrition and immunity reported in lines 67-76. This information is not relevant because your study is about the commitment and adherence to the preventive measures and nutrition is not one of the measures. Could you please remove this text along with the references cited in this paragraph?

**Author response:** We agree with the editor, and have removed this paragraph from the manuscript. These amendments have been made to the manuscript text and where these can be viewed in the track change manuscript (Background, line 70-79)

3- Please change ‘6pm-to-6am’to ‘6 pm-to-6 am’ (line 79)

**Author response:** We apologize for this mistake and have made the asked modifications. (Background, line 82)

4- Could you please change ‘wearing masks’ to ‘wearing face masks’ (line 83).

**Author response:**  We apologize for this mistake and have made the asked modifications. (Background, line 87)

5- Could you please report the exact date instead of writing: ‘As of this writing’ in the following sentence ‘As of this writing, 45,468 laboratory-confirmed cases and 2,637 casualties.. (Line 88).

**Author response:** We understand the editor’s point, and have mentioned the exact date for these figures. These amendments have been made to the manuscript text and where these can be viewed (Background section, line 91-92)

METHODS:

6- Study design, setting, and participants: Please report your total sample size (how many people were invited /approached to complete the survey questionnaire) because there are different numbers reported in the manuscript. For example, in the abstract sample size is ‘10083’ reached out’ while in the methods section you report calculated sample size = 7336 and then you report 8083 people completed the survey and thereafter ‘final sample size of 7,531 participants. Please clearly report your calculated sample size as well as how many people were invited/reached out (if there is difference between these then give reasons for it). Then how many responses were received and how many cases were excluded and final sample size / responses that were analysed. In the abstract you report your final sample size included in the analysis as well as effective response rate.

**Author response:** We understand the editor’s point and would like to clarify that initially the sample size calculation software suggested a minimum sample size of 7336. In order to achieve this goal, we reached out to 10083 individuals, and of these only 8083 responded back to us. After reviewing these responses we excluded those who did not meet our inclusion criteria, which eventually gave us a final figure of 7531. This clarification was made to the manuscript where it can be viewed (Method section, line 124- 132)

7- Could you please provide more information about how your sample was ‘nationally representative sample’? (Line 110).

**Author response:** We thank the editor for this comment, and have removed this statement from the methods section as it is fully explained later on the discussion section. These amendments have been made to the manuscript text and where these can be viewed (Method section, line 113)

8- You report ‘Chain-referral and convenience sampling methods were employed...’. These might include ‘snowball sampling’? If so, please report it here as well as in the abstract.

**Author response:** We agree with the editor, and yes indeed, snowball sampling is a synonym for chain-referral sampling. We added this term appropriately in the method section where these can be viewed (methods section, line 116)

9- Please refer to you statement: ‘The questionnaire, based on a previous study,..’ (Line 110). Could you please provide a reference/cite this study that used this questionnaire originally?

**Author response:** We thank the editor for the guidance. However, the questionnaire we used is a newly developed one based on multiple different questionnaires from multiple previous studies (references 15-17). We modified this sentence and made this idea clear which can be viewed in (Method section, line 113-114)

10- Could you please report in the methods section how financial status was categorised because Table 5 includes categories of the financial status of the participants

**Author response:** We thank the editor for the guidance. We mentioned the categories in the method section as required (Methods section, line139-140)

11- Statistical analysis: Could you please add the abbreviation of names of the researchers who manually entered data in the following sentence: ‘Data from the hard copy questionnaires were entered manually by the investigators (AB, XY, etc) (lines 149-150).

**Author response:** We agree with the editor, and have added the initials of the authors who manually entered the data. These amendments have been made to the manuscript text and where these can be viewed (Methods section, line 155-156).

12- ‘A 13 point scale was used to measure the level of commitment (Line 152) was this scale created by the authors of this study or it was already reported in the original questionnaire adopted from earlier studies (which ones?).

**Author response:** We understand the editor’s point and would like to clarify that the scale was developed by the investigators for this study. We also clarified this point in the manuscript. These amendments have been made to the manuscript text and where these can be viewed (Method section, line 158-159)

13- Please report the lowest and highest scores in the 13-point scale.

**Author response:** We thank the editor for the guidance. We added the lowest and highest scores in the 13-point scale (Methods section, line160)

14- Ethics & Consent: The authors report that ‘Informed consent was obtained from every participant…’ (line 164). Could you please report how consent from participants was obtained and in which form it was obtained?

**Author response:** We thank the editor for the guidance. We clarified that a written informed consent was obtained from every participant as each questionnaire had an informed consent form (the first page in the hard copy version and the first question in the digital one) needs to be signed by the respondents prior to participation. (Methods section, line171 - 173)

RESULTS:

15- Participant characteristics: The authors report that ‘Of 10083 participants, 7531 agreed to participate,’ (Line 170) whereas in the methods section they report that 8083 people completed the survey (Line 123). Please report the actual number of participants who completed the survey and then how many surveys were fully completed. You might like to say: ‘Of 8083 retuned surveys, 7531 surveys were fully completed and analysed’. Then you can report your affective response rate

**Author response:** We thank the editor for the guidance. We addressed this point and made it clear in both results and Methods sections. (Methods section, line1791 - 181)

16. Participant characteristics: Please avoid reporting same information in the text as well as in Tables. For example: ‘…3505 (46.5%) were males and 4026 (53.5%) were females. 172 The dominant age group was 18-24 years old: 3124 (41.5%), followed by 25 - 44 years old: 2338 173 (31%), 45-65 years old: 1686 (22.4%), and > 65 years: 383 (5.1%).’ (Lines 171-173. Here your repot counts and % of items/variables that are also given in Table 1. Report only the main / major findings in the text and refer to table for numbers and %. For example: Most of the respondents were women and the dominant age group was 18-24 years (Table 1).
17. Please revise this sentence: Over the half of the study population originated from the central governorates: 4376 (58.1%), and 1165 (15.5%) from the western governorates. (Lines 174-175). You might like to report as: Just more than half of the respondents originated from the central governorates while the lowest respondents were from the western governorates.
18. Please revise the text reported in lines 176-181 as suggested above.
19. Please revise the text about the findings about ‘COVID-19 related information’ and ‘Commitment to preventive measures’ as suggested in comments 15-16 above.

**Author response:** We thank the editor for the guidance. We addressed this point were appropriate across all result sections.

20. Please revise ‘…showed poor commitment. (Table 3).’ As ‘...showed low commitment. (Table 3).’ (Line 201).

**Author response:** We thank the editor for the guidance. We addressed this issue across all result sections.

21. Table 3: In this table commitment levels are reported as ‘Bad, Moderate, and Good’, whereas in the methods section commitment levels reported are: ‘Low’, ‘Moderate’ and ‘High’ (lines 155-156). Please be consistent and report the level of commitment as reported in the methods section.

**Author response:** We thank the editor for the guidance. We addressed this issue across all result sections.
22. Table 4, Please start each statement with a capital letter i.e. wearing as Wearing, cancelled as Cancelled and so on in this Table.

**Author response:** We thank the editor for the guidance. We addressed this issue and corrected all statements.
23. P-values: Please report p-values as <0.001 for all values 0.000 or <0.0001 in the section about ‘Correlations between commitment to preventive measures and participants characteristics’ (lines 212-233), Table 5, Table 6 and elsewhere.

**Author response:** We thank the editor for the guidance. We addressed this issue across the manuscript

24. Table 5 & Table 6: Please revise ‘Bad’ to ‘Low’ in column ‘Level of commitment’ in these tables and elsewhere.

**Author response:** We thank the editor for the guidance. We addressed this issue across the manuscript

DISCUSSION

25. Please remove the text in the first paragraph in lines 240-248 “In talking about infectious diseases,…………. also removed and become self-limiting (28).” This information is about issues that are different from the focus of this study which is about the commitment to preventive measures to prevent the spread of COVID-19. Please remove all references (23-27) cited in this para.

**Author response:** We agree with editor’s point of view and have removed this paragraph and the corresponding references.

26. The authors argue that ‘This level of adherence is similar to that found in a Belgian study’; however, comparison between Belgium and Syria does not seem to be reasonable because the two countries are very different in many aspects social, economic and so on. The authors might like to revise this argument. Similarly in the following sentence the authors compare their findings with the UK and Germany also. The authors should compare their findings with the countries in their region and with similar socio-economic situation.

**Author response:** We understand the editor’s point of view and we would like to clarify that we compared our results with results from countries with different ranking because we believe that it is important to understand our country’s situation compared to the world.

27. Please change ‘The majority of our sample were young...’ (Line 261) to ‘The majority of our participants were young…’

**Author response:** We thank the editor for the guidance and have made the required modification (Discussion section, line 273)

28. The authors report that ‘Low income and unemployment were correlated with lower adherence to IPC measures, while higher income and gainful employment was correlated with higher adherence’ (Lines 273-274). Table 5 shows similar findings about the low education level and vice versa. The authors could include the findings about the education level in this sentence. These findings are important from the policy perspective and could be included/highlighted in the conclusion.

**Author response:** We thank the editor for the guidance. However, we talked about the correlation between educational level and commitment to protective measures later in the discussion with its own paragraph (Discussion section, line 303-312)

CONCLUSIONS
29. Conclusion reported in the abstract and in the main manuscript should convey the same message so please revise your conclusion at both places.

**Author response:** We thank the editor for the guidance. We modified the conclusion in the abstract to convey the main message as the main conclusion.
