# Supplementary material for: Commitment to protective measures during the COVID-19 pandemic in Syria: A nationwide cross-sectional study
Source: PLoS One. 2022 Oct 14;17(10):e0275669. doi: 10.1371/journal.pone.0275669 (PMC9565665; doi:10.1371/journal.pone.0275669)
Supplement: Attachment — Submitted filename: Response to Reviewers.docx [file pone.0275669.s004.docx]

Response letter

**We would like to thank the editors for taking their valuable time to review this manuscript. We have thoroughly assessed the comments and implemented them into our revised manuscript, now that this article has been made suitable we aspire to have this article published in your journal.**

**The following letter will report the responses to the reviewers’ comments.**

**Comments from the Academic Editor:**

**1.** Authorship: A new author named ‘Dana Alakhrass’ with affiliation no. 6 has been added in this version of the manuscript. This person was not a author in the original and revised submission R1. This person could not be added as an author at this stage. If this person helped in the latest revision of the manuscript, then their name could be reported in the acknowledgments. Please refer to the PLOS ONE Guidelines for the authorship for further guidance on the issue.

**Author response:** We apologize for this mistake and have removed this person name from the authorship list to the acknowledgment section These amendments have been made to the manuscript text and where these can be viewed (acknowledgment section, lines 332-333)

**2.** Abstract; Conclusion- Please change 'a nationwide awareness campaigns ' to either 'A nationwide awareness campaign' or 'Nationwide awareness campaigns' in the conclusion in the abstract.

**Author response:** We thank the academic Editor for the guidance, and have changed 'a nationwide awareness campaigns ' to 'Nationwide awareness campaigns'. These amendments have been made to the manuscript text and where these can be viewed (Abstract section, lines 48-49)

**3.** Background: Lines 58-59. Please correct the following sentence 'to the date of writing in December 2021'. It could be ‘as of XX December 2021' (put a particular date instead of to the date of writing).

**Author response:** We thank the AE for the comment, and have added the exact date corresponding with the numbers to the manuscript. These amendments have been made to the manuscript text and where these can be viewed (Introduction section, lines 56-57)

**4.** Methods: Lines 115-116, Please change from 'based on a previous studies' to 'based on previous studies'

**Author response:** We apologize for this mistake and have revised the sentence. These amendments have been made to the manuscript text and where these can be viewed (methods section, line104)

**5.** Financial status in Table 1 and other text: Please change financial status category of 'bad' to 'low', which reads better. Make this change in all places where you have reported 'bad' financial status.

**Author response:** We thank the AE for the comment, and have changed the financial status category of 'bad' to 'low' throughout the manuscript.

**6.** Table 4: Specific preventive measures: Some of these statements start with a past tense while other use different tense. Could you please correct these statements so that they are in the same tense? All these statements should start with a capital letter.

**Author response:** We thank the academic Editor for the guidance, and have changed the tenses of all statements to be in the past tense. These amendments have been made to the manuscript text and where these can be viewed (Table 4)

**7.** Table 4: What do you mean by the amount in the following sentence 'Reduce the amount you go to shops'? Is it the 'time' or 'number of times' or something else? Please change accordingly.

**Author response:** We apologize for this mistake and have clarified the exact meaning of the question by changing 'Reduce the amount you go to shops'? to 'Reduce the number of times you go to shops'? . These amendments have been made to the manuscript text and where these can be viewed (Table 4.)

**8.** Legends of figures and table captions: Please put full stop after the number. Change from Table .1 to Table 1. Please do the same for all figures and tables.

**Author response:** We apologize for this mistake and have applied the required amendments. (Tables and figures legends section, lines 335-342)

**9**. References: please update your references and include the vol. issue and page numbers as well as DOI for all journal articles and URLs for internet / website sources. In some reference year is written twice, please report it only once.

**Author response:** We have updated the references list to include the vol. issue and page numbers as well as DOI for all journal articles and URLs for internet / website sources.
